# Supplementary material for: Vitamin D Modified DSS-Induced Colitis in Mice via STING Signaling Pathway
Source: Biology (Basel). 2025 Jun 18;14(6):715. doi: 10.3390/biology14060715 (PMC12190092; doi:10.3390/biology14060715)
Supplement: Supplementary file 1 [file biology-14-00715-s001.zip › Supplementary Materials S1: 16s-report of gut microbiota/Groups/betadiv/pcoa_unweighted_unifrac_analysis2/Pcoa_unifrac12_boxplot_ellipse.pdf]

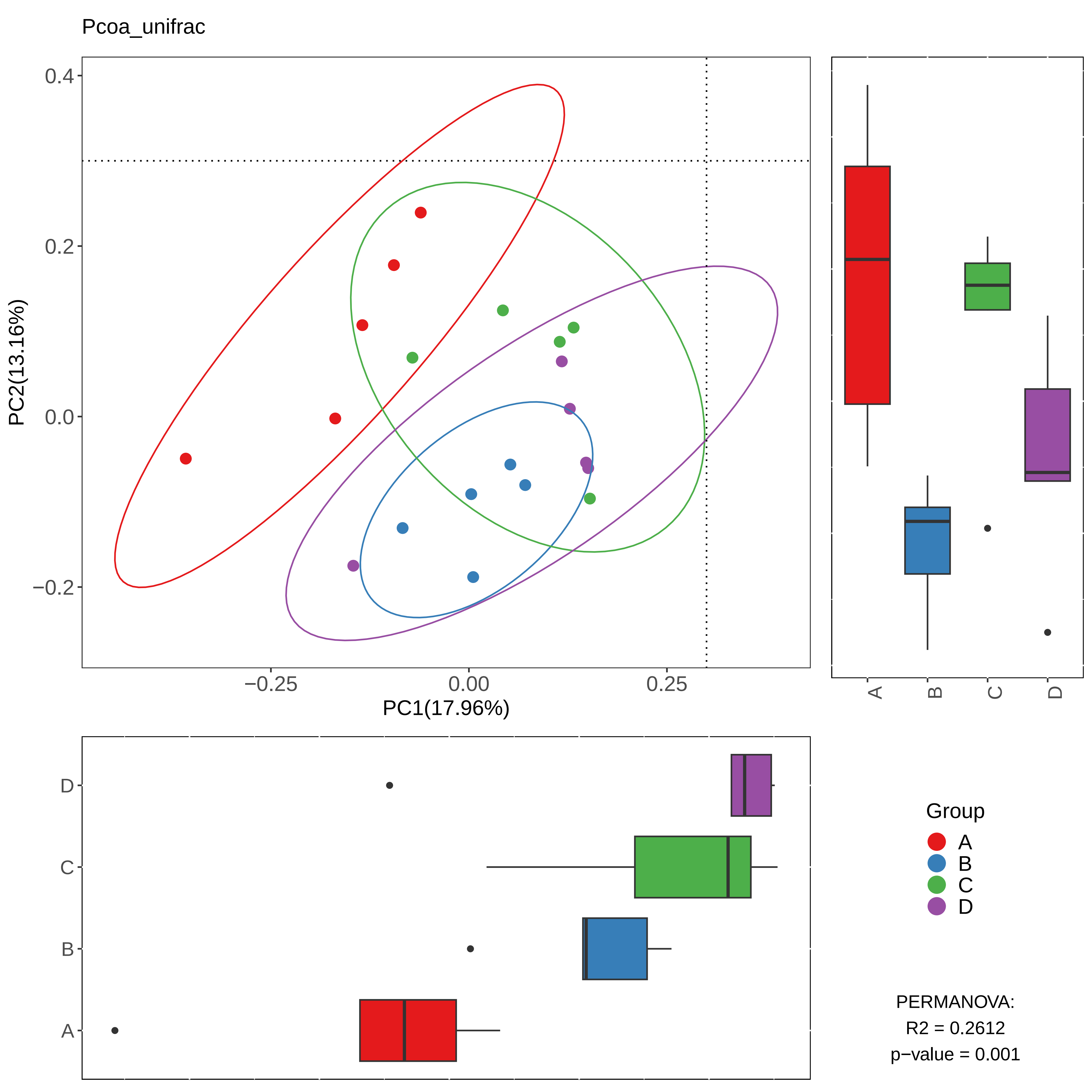

Pcoa_unifrac
0.4
0.2
0.0
−0.2
PC2(13.16%)
−0.25
0.00
0.25
PC1(17.96%)
A
B
C
D
D
C
B
A
Group
A
B
C
D
PERMANOVA:
R2 = 0.2612
p−value = 0.001
